# Human Cytomegalovirus UL18 Utilizes US6 for Evading the NK and T-Cell Responses

**DOI:** 10.1371/journal.ppat.1000123

**Published:** 2008-08-08

**Authors:** Youngkyun Kim, Boyoun Park, Sunglim Cho, Jinwook Shin, Kwangmin Cho, Youngsoo Jun, Kwangseog Ahn

**Affiliations:** National Creative Research Initiatives Center for Antigen Presentation, Department of Biological Sciences, Seoul National University, Seoul, Korea; University of Southern California School of Medicine, United States of America

## Abstract

Human cytomegalovirus (HCMV) US6 glycoprotein inhibits TAP function, resulting in down-regulation of MHC class I molecules at the cell surface. Cells lacking MHC class I molecules are susceptible to NK cell lysis. HCMV expresses UL18, a MHC class I homolog that functions as a surrogate to prevent host cell lysis. Despite a high level of sequence and structural homology between UL18 and MHC class I molecules, surface expression of MHC class I, but not UL18, is down regulated by US6. Here, we describe a mechanism of action by which HCMV UL18 avoids attack by the self-derived TAP inhibitor US6. UL18 abrogates US6 inhibition of ATP binding by TAP and, thereby, restores TAP-mediated peptide translocation. In addition, UL18 together with US6 interferes with the physical association between MHC class I molecules and TAP that is required for optimal peptide loading. Thus, regardless of the recovery of TAP function, surface expression of MHC class I molecules remains decreased. UL18 represents a unique immune evasion protein that has evolved to evade both the NK and the T cell immune responses.

## Author Summary

HCMV establishes a lifelong latent infection and causes serious disease in immunocompromised individuals. Cytotoxic T lymphocytes (CTL) and natural killer (NK) cells are the primary effectors for the immune defense against HCMV. However, HCMV has evolved to evade both the innate and adaptive cellular immunity to viral infection. HCMV US6 glycoprotein inhibits TAP function, resulting in down-regulation of MHC class I, while HCMV UL18 is an MHC class I homolog that functions as a surrogate to prevent host cell lysis. Despite significant sequence and structural homology between UL18 and MHC class I molecules, US6 down regulates surface expression of MHC class I, but not UL18. Here, we describe a mechanism by which UL18 circumvents the self-derived TAP inhibitor, US6. UL18 abrogates US6 inhibition of TAP-ATP binding and restores TAP-mediated peptide translocation, thereby making peptides available for the assembly and subsequent surface expression of UL18. Together UL18 and US6 inhibit binding of MHC class I to TAP, thus down regulating surface expression of MHC class I molecules. UL18 represents a unique immune evasion protein resistant to both the NK and T cell immune responses. Our data provide a molecular basis for persistent HCMV infection and will aid in the development of a therapeutic vaccine.

## Introduction

Human cytomegalovirus (HCMV), a β-herpesvirus, is prevalent in human populations worldwide [1]. Commonly, HCMV infection remains in a chronic, latent state. However, HCMV reactivation can result in morbidity or death in immunocompromised individuals, such as organ transplant recipients or AIDS patients [2]. MHC class I-restricted CD8^+^ cytotoxic T lymphocytes (CTL) play a major role in controlling viral infections. CTLs recognize and lyse virus-infected cells through engagement of the T cell receptor with MHC class I molecules presenting viral antigens at the surface of infected cells [3].

Newly-synthesized MHC class I heavy chain associates with β_2_-microglobulin (β_2_m) to form a heterodimer. This heterodimer is recruited into the MHC class I peptide-loading complex, which consists of MHC class I, β_2_m, TAP, calreticulin, ERp57, tapasin, and protein disulfide isomerase [4],[5], that controls the optimal peptide loading into the peptide-binding groove of MHC class I molecules. Only peptide-loaded class I molecules exit from the endoplasmic reticulum (ER) for transport to the cell surface. Peptides are generated primarily in the cytosol by proteasomes and then translocated to the ER by TAP for binding to MHC class I molecules [6],[7]. The TAP1/TAP2 heterodimer is a member of the ATP-binding cassette transporter superfamily and utilizes energy from ATP hydrolysis to translocate peptides into the lumen of the ER [8].

With selective pressure from the host immune response, HCMV has evolved several gene products which interfere with antigen presentation and eventual cell surface expression of MHC class I molecules. HCMV encodes four individual gene products of the unique short region protein (US): US2, US3, US6, and US11. Each protein is independently able to reduce class I surface expression [9]. For example, US6 is an ER-resident glycoprotein that binds directly to TAP in the ER lumen [10],[11]. US6 blocks the binding of ATP by TAP1 through a conformational change and subsequently inhibits TAP-mediated peptide translocation to the ER [12].

Although interference in antigen presentation and consequent MHC class I down-regulation on the cell surface might allow infected cells to evade virus-specific CTL, down-regulation of MHC class I molecules makes these cells susceptible to lysis by NK cells, which target cells lacking MHC class I molecules [13]. HCMV encodes multiple genes that function to evade NK-mediated cell lysis of infected cells by distinct mechanisms [14]–[16], suggesting that NK cells are crucial components of the innate defense against HCMV. UL18 is a MHC class I homolog that acts as a decoy to block NK cell cytotoxicity [17]. UL18 binds LIR-1, an NK cell inhibitory receptor, with an affinity exceeding that of MHC class I to LIR-1 by greater than 1000-fold [18]. UL18 is a type I transmembrane glycoprotein that shares a high level of amino acid sequence identity with MHC class I [19],[20]. Like MHC class I molecules, UL18 can associate with β_2_m [21] and bind endogenous peptides that are similar to peptides loaded on an MHC class I molecule [22]. Surprisingly, despite the sequence and structural similarities between MHC class I molecules and UL18 [20], US6 down regulates only MHC class I and not UL18 [23].

Here, we describe a novel mechanism of action for UL18 that may account for the specific down-regulation of one homolog and not the other. UL18 restores the peptide transport activity of TAP by inactivating US6. In addition, UL18 impairs optimal peptide binding by MHC class I molecules by interfering with the assembly of the peptide-loading complex. Hence, even though TAP function is recovered, the surface level of MHC class I molecules remains down regulated. Our data provide insight into how the viral MHC class I homolog UL18 has effectively evolved to evade both NK and CTL immune responses.

## Results

### UL18 is dependent on TAP for cell surface expression but is insensitive to the TAP inhibitor US6

**Figure 1:**
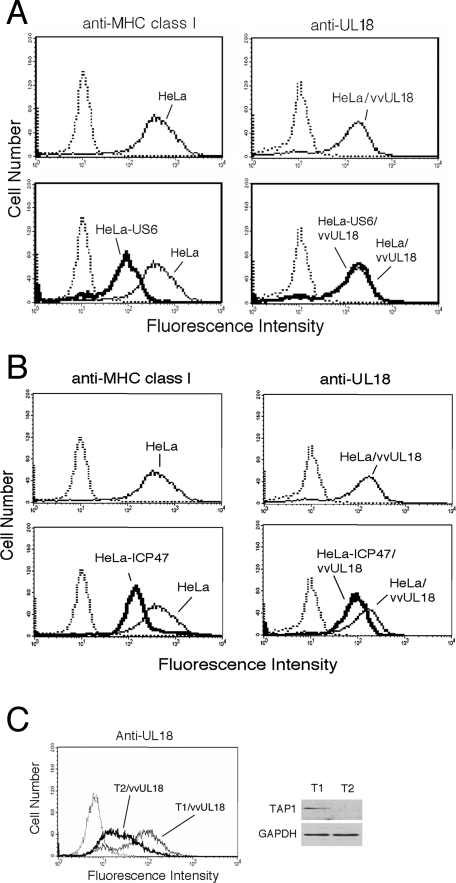
Cell surface expression of UL18 was TAP-dependent. HeLa, HeLa-US6, and HeLa-ICP47 cells were infected with either vvUL18 or vvWT. Cells were preincubated with mAb W6/32 (anti-MHC class I), 10C7 (anti-UL18), or normal mouse IgG (negative control) and stained with FITC-conjugated anti-mouse IgG. Fluorescence profiles are shown. Dashed lines indicate the negative control. (*A*) The surface expression of UL18 was not influenced by US6, the self-TAP inhibitor. (*B*) The surface expression of UL18 was down regulated by ICP47, the HSV-derived nonself- TAP inhibitor. (C) Down-regulation of UL18 surface expression in the T2, TAP-deficient cell line. T1 was included as a TAP-positive control. Representative FACS plots are shown.

We previously reported that unlike MHC class I expression, cell surface expression of HCMV UL18 is resistant to the self-derived TAP inhibitor US6 [23]. Considering both the temporal coexistence of the UL18 and US6 gene products during infection and sequence similarities between MHC class I and UL18, we raised the intriguing question of how UL18 avoids ‘self attack’ by US6. We first confirmed that US6 differentially regulates surface expression of MHC class I and UL18. We infected HeLa cells stably expressing US6 (HeLa-US6) or parental HeLa cells with either a recombinant UL18-expressing vaccinia virus (vvUL18) or a wild-type vaccinia virus (vvWT). To measure cell surface levels of MHC class I and UL18, we used the monoclonal antibodies (mAbs) W6/32 and 10C7 and flow cytometry analysis. Consistent with our previous observation [23], US6 down regulated the surface expression of MHC class I molecules (Fig. 1*A*, left column), but did not influence surface levels of UL18 (Fig. 1*A*, right column).

To date, the possible dependence of UL18 on TAP for surface expression has not been confirmed. To address this question, we assessed the effect of ICP47, the herpes simplex virus-derived TAP inhibitor [24],[25], on the surface expression of UL18. In contrast to US6, ICP47 binds to the peptide-binding site on the cytosolic side of TAP, thereby, blocking transport of peptide ligands into the ER [26],[27]. As expected, the surface level of MHC class I molecules was down regulated in cells stably expressing ICP47 (HeLa-ICP47) (Fig. 1*B*, left column). The surface level of UL18 was also reduced in HeLa-ICP47 cells (Fig. 1*B*, right column) unlike in HeLa-US6 cells, indicating the TAP-dependency of UL18 for surface expression. Surface levels of UL18 were significantly reduced in TAP-deficient mutant T2 cells compared to normal T1 cells (Fig. 1*C*), further supporting the dependence of UL18 on TAP for surface expression.

### UL18 counteracts US6-mediated inhibition of TAP

**Figure 2:**
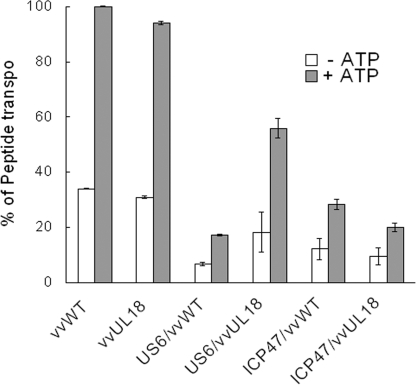
Peptide transport inhibited by US6 was restored upon expression of UL18. HeLa, HeLa-US6, and HeLa-ICP47 cells were infected with either vvWT or vvUL18. Cells were permeabilized with Streptolysin O and incubated with the FITC-conjugated peptides in the presence (filled bars) or absence of ATP (open bars). Transported peptides were quantified by fluorescence measurements (excitation and emission at 485 and 520 nm, respectively). The y-axis reflects peptide transport expressed as a percentage of translocation relative to the translocation observed in control cells. Averages and S.D. were calculated from three independent experiments.

The above data suggest that UL18 modulates the inhibitory action of US6 on TAP activity. To test this hypothesis, we directly measured TAP-dependent peptide transport in cells expressing either UL18 alone, both UL18 and US6, or both UL18 and ICP47. A significant reduction in peptide translocation was observed in cells expressing the US6 protein alone (HeLa-US6) and cells expressing ICP47 alone (HeLa-ICP47). Expression of only the UL18 protein did not affect peptide transport. Interestingly, upon ectopic expression of UL18 in HeLa-US6, peptide translocation into the ER lumen was markedly restored (Fig. 2), suggesting that UL18 attenuates the inhibitory effect of US6 on TAP. UL18-induced recovery of TAP function was not observed in cells expressing ICP47 (Fig. 2). The peptide transport results correlate with the cell surface expression of UL18 molecules (Fig. 1*A*).

### Molecular basis for reversal of US6-mediated inhibition of TAP by UL18

**Figure 3:**
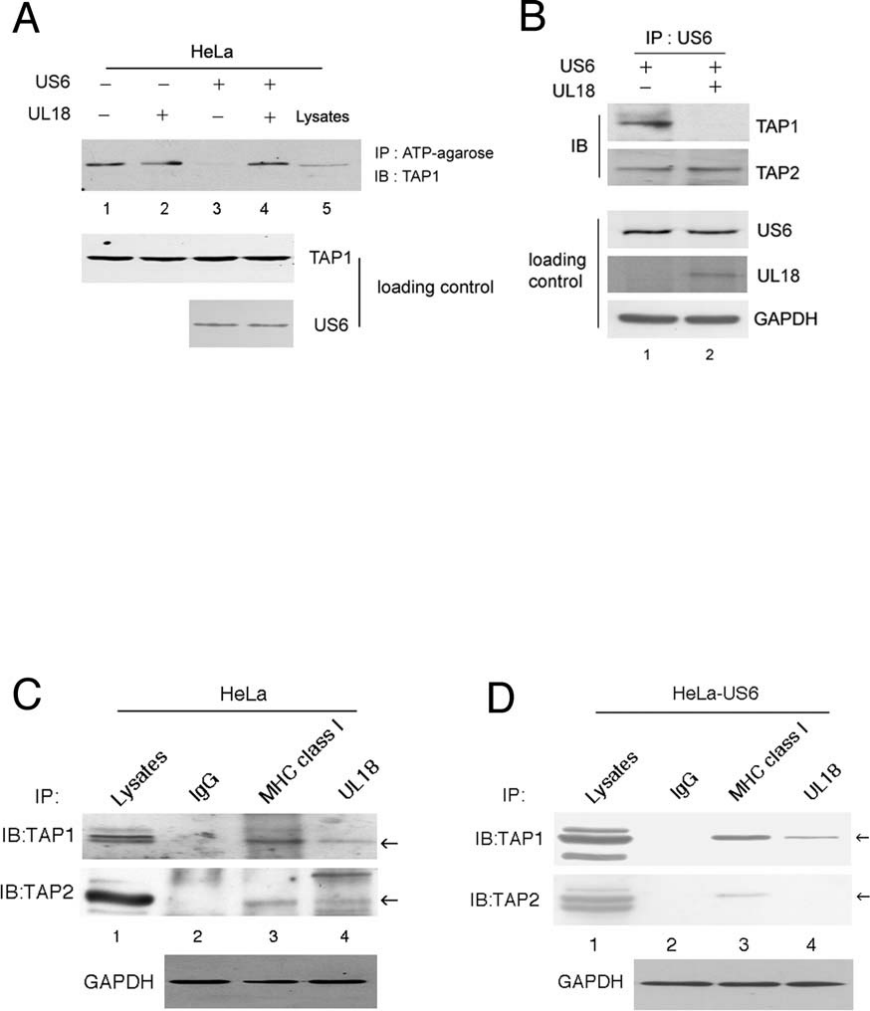
Molecular mechanism for the reversal of US6-mediated TAP inhibition by UL18. (*A*) UL18 restored ATP binding by TAP. HeLa and HeLa-US6 infected with either vvWT or vvUL18 were extracted in 1% digitonin. Digitonin lysates were incubated with N-6 ATP-agarose for 1 h, and agarose-bound proteins were separated by 10% SDS-PAGE and analyzed by immunoblotting with TAP1-specific antibodies. As a loading control, aliquots of cell lysates were probed with TAP1 and US6-specific antibodies. (*B*) UL18 interfered with the association between US6 and TAP1. HeLa-US6 cells were infected with either vvWT or vvUL18, lysed in 1% digitonin, and immunoprecipitated with anti-US6 antibodies. The US6 immunoprecipitates were separated by 8% SDS-PAGE and probed with polyclonal TAP1 and TAP2 antibodies. Cell lysates were subjected to immunoblotting with anti-US6 and immunoprecipitation with anti-UL18 to verify the expression of US6 and UL18. GAPDH was included to serve as a loading control. (C) UL18 bound to both TAP1 and TAP2. HeLa cells infected with vvUL18 were lysed in 1% digitonin and immunoprecipitated with either anti-UL18 mAb, 10C7, or anti-MHC class I polyclonal Ab, H-300 followed by blotting with TAP1 antibody (top panel) and TAP2 antibody (middle panel). GAPDH was included to verify equal loading of proteins (bottom panel). (D) US6 inhibited the association of UL18 with TAP2. Identical analysis was performed as described in Figure 3*C*, except for the use of HeLa cells stably expressing US6. Data are representative of three independent experiments with similar results.

Peptide translocation by TAP occurs in two steps: peptide binding to TAP and peptide translocation involving ATP binding and hydrolysis [28],[29]. US6 inhibits TAP by preventing ATP from binding to TAP1 [12]. To examine whether UL18 affects the ATP binding of TAP1 inhibited by US6, we compared the ATP-binding capacity of TAP in HeLa-US6 with that in HeLa-US6 expressing UL18. Cells were lysed and incubated with ATP-agarose. Proteins bound to the ATP-agarose were eluted, separated by SDS-PAGE, and probed with anti-TAP1 antibody. Consistent with the results of the previous study [12], US6 inhibited ATP binding by TAP1 (Fig. 3*A*, lane 3). UL18 alone did not interfere with ATP binding of TAP1 (Fig. 3*A*, lane 2). Interestingly, inhibition of ATP binding to TAP1 by US6 was abolished upon expression of UL18 (Fig. 3*A*, lane 4), demonstrating that UL18 restores the ability of TAP to bind ATP. To further define the mechanism underlying the reversal of US6-mediated inhibition of ATP binding to TAP, we investigated the effect of UL18 on the physical association between US6 and TAP that is required for inhibition of TAP by US6 [11]. HeLa-US6 cells were infected with vvWT or vvUL18 and lysed in 1% digitonin, and cell lysates were coimmunoprecipitated with anti-US6 antibody. Anti-US6 immunoprecipitated proteins were resolved by SDS-PAGE, followed by immunoblotting with antibodies. UL18 disrupted the interaction between US6 and TAP1 while not affecting the association of US6 with TAP2 (Fig. 3*B*, first and second panels). Therefore, we conclude that UL18 induces the dissociation of US6 from TAP1, restoring TAP-mediated peptide transport.

How does UL18 specifically block the binding of US6 to TAP1 but not to TAP2? To address this question, we examined the interactions among MHC class I, TAP, and UL18 in UL18-expressing HeLa and HeLa-US6 cells. Coimmunoprecipitation and western blot analysis indicated that MHC class I molecules bind to both TAP1 and TAP2 irrespective of US6 (Fig. 3*C*, lane 3 and Fig. 3*D*, lane 3). In the absence of US6, UL18 also interacted with both TAP1 and TAP2 (Fig. 3*C*, land 4). Upon US6 expression, however, the interaction of UL18 with TAP2 was virtually undetectable, while its interaction with TAP1 remained unaffected (Fig. 3*D*, upper and lower panels, lane 4). Taken together, these results demonstrate that US6 binds more competitively to TAP2 than does UL18, while UL18 more competitively binds to TAP1.

### Recovery of TAP function by UL18 is not sufficient to restore cell surface expression of MHC class I

**Figure 4:**
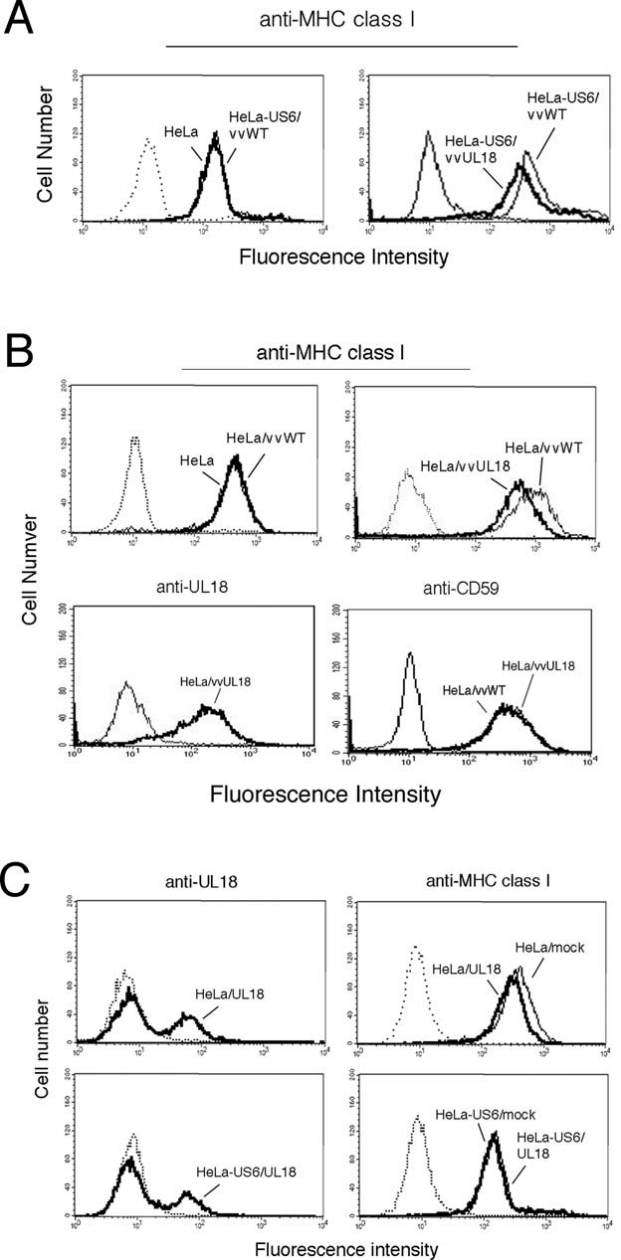
The recovery of TAP function by UL18 was not sufficient to restore the cell surface expression of MHC class I. (*A*) The surface expression of MHC class I molecules was not restored despite the recovery of TAP activity mediated by UL18 and US6. HeLa-US6 cells were infected with vvUL18 and then analyzed for MHC class I surface expression by flow cytometry after preincubation with mAb W6/32. (*B*) Specific down-regulation of MHC class I surface expression by the vaccinia virus- expressed UL18. HeLa cells were infected with vvUL18, stained for 1 hr with the indicated antibodies, and analyzed by FACS. *C*) Down-regulation of MHC class I molecules by UL18 expressed by a transient transfection system. HeLa and HeLa-US6 cells were transfected with either mock vector or UL18 cDNA. Cells were analyzed by FACS using mAbs 10C7 and W6/32 as described in Figure 4*C*. The results are representative of three independent experiments.

Because the peptide supply was recovered upon coexpression of US6 and UL18, we expected that the surface level of MHC class I molecules would increase to the steady-state level in normal cells. However, surface levels of MHC class I molecules were not recovered (Fig. 4*A*). Expressing UL18 in HeLa-US6 cells further down regulated surface levels of MHC class I molecules when compared to those of parental HeLa-US6 cells (Fig. 4*A*, right), suggesting that down-regulation of MHC class I by UL18 and US6 might be additive. Next, we tested whether UL18 alone inhibits MHC class I surface expression. In the presence of UL18, we observed consistent down-regulation of the surface expression of MHC class I (Fig. 4*B*, upper right panel), albeit at significantly reduced efficiency relative to the extent of class I down-regulation observed in cells expressing US6 alone (Fig. 4*A*, left panel). As a control, the cell surface level of CD59 was not affected in the same cells (Fig. 4*B*, lower right panel), confirming that the specific effect of UL18 on the surface expression of MHC class I. Similar results were obtained using a transient transfection system. In agreement with a recent report that low levels of UL18 are detectable on the surface of UL18-transfected HeLa cells [30], we also detected transiently expressed-UL18 at the surface of HeLa cells (Fig. 4*C*, upper left panel). More importantly, surface levels of MHC class I molecules were reduced slightly upon transient expression of UL18 (upper right panel), similar to the vaccinia virus system. This result is consistent with the observation that surface levels of MHC class I are slightly increased in cells infected with HCMV AD169 mutant with the UL18 gene deleted [31]. Surface levels of MHC class I molecules were not recovered in HeLa-US6 cells transiently expressing UL18, as was the case with the vaccinia virus system (lower right panel). These results further eliminate the possibility that results obtained using vvUL18 are an over expression artifact in the vaccinia virus infection system.

### UL18 impairs optimal peptide loading by MHC class I molecules

**Figure 5:**
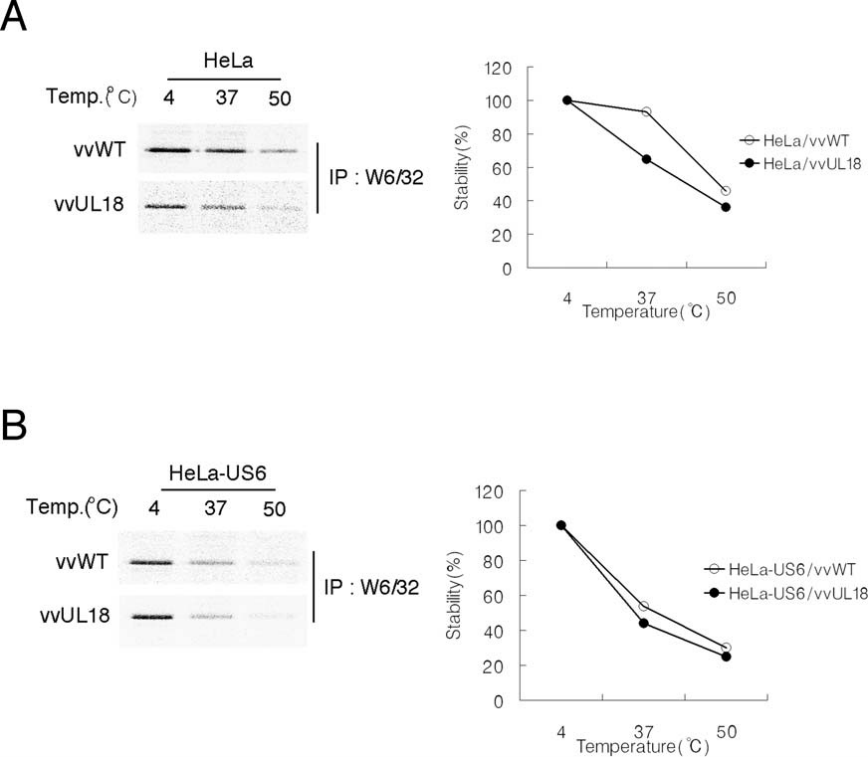
UL18 impaired optimal peptide loading of MHC class I molecules. HeLa (*A*) and HeLa-US6 cells (*B*) infected cells with either vvWT or vvUL18 were radiolabeled for 30 min and then lysed in 1% NP-40 lysis buffer. Equal lysate aliquots were incubated at 4°C, 37°C, or 50°C for 40 min prior to immunoprecipitation with mAb W6/32. For each temperature, the intensity of the MHC class I heavy chain radioactive band was quantified. Similar results were obtained in three independent experiments.

To identify the molecular mechanisms for down-regulation of MHC class I molecules by UL18, we investigated the effect of UL18 on optimal peptide binding by MHC class I molecules. We assessed the extent of optimal peptide binding by an established assay that correlates thermostability with the affinity of MHC class I peptide cargo [32]. Radiolabeled detergent lysates of HeLa and HeLa-US6 cells infected with either vvWT or vvUL18 were incubated at 4°C, 37°C, or 50°C prior to immunoprecipitation with mAb W6/32. In vvUL18-infected HeLa cells relative to vvWT-infected cells, MHC class I molecules were less thermostable at all temperatures, with the most prominent difference observed at 37°C (Fig. 5*A*). In HeLa-US6 cells, we did not detect differences in thermostability in the absence or presence of UL18 (Fig. 5*B*). The lack of significant UL18 influence on thermostability in HeLa-US6 cells is presumably due to the overwhelming effect of US6. Collectively, our data suggest that UL18 inhibits optimal peptide loading of MHC class I molecules and subsequently down regulates surface expression of MHC class I molecules.

### UL18 inhibits the interaction between MHC class I and TAP1

**Figure 6:**
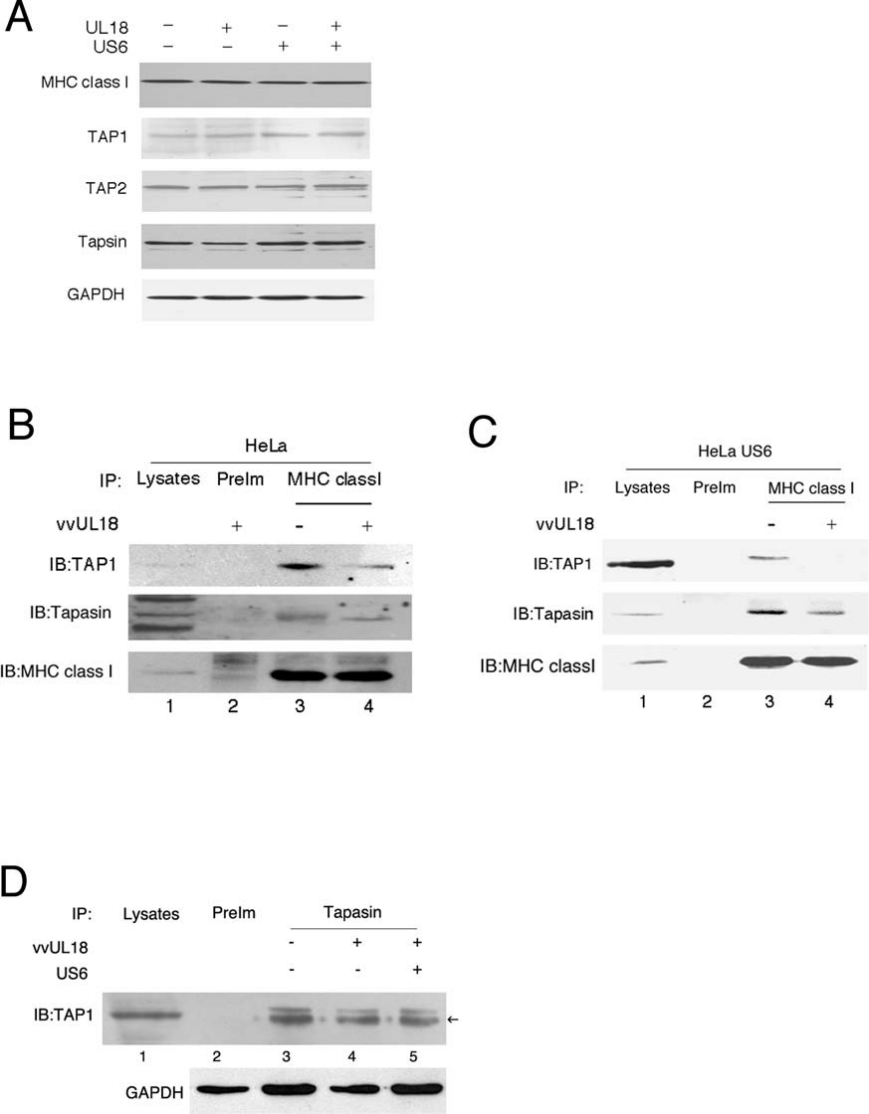
UL18 interfered with the assembly of the peptide-loading complex. (*A*) The expression level of the peptide-loading complex components was not affected by UL18 or US6. HeLa (−US6) and HeLa-US6 cells (+US6) infected with either vvUL18 or vvWT were lysed with 1% NP-40 Whole cell lysates were subjected to western blot analysis with the antibodies specific to MHC class I, TAP1, or tapasin. GAPDH served as a loading control. (*B*) UL18 inhibited the MHC class I-TAP1 and MHC class I-tapasin interactions. Infected cells were lysed in 1% digitonin and immunoprecipitated with anti-MHC class I antibody. Eluted proteins from the immunoprecipitates were resolved by SDS-PAGE, and the blots were probed with anti-TAP1 (top panel) and anti-tapasin antibody (middle panel). The same blot was stripped and reprobed with anti-MHC class I antibody as a loading control (bottom panel). (*C*) In the presence of US6, UL18 disturbed the association between TAP1 and MHC class I. Analysis was performed as described in Figure 6*B*, except HeLa-US6 cells were used. (*D*) Neither UL18 nor US6 affected the interaction between tapasin and TAP1. HeLa (−) or HeLa-US6 cells (+) were infected with vvUL18. Whole cell lysates of 1% digitonin were immunoprecipitated with anti-tapasin antibody, and blots were probed with anti-TAP1 antibody. Data (*A–D*) are representative of at least three independent experiments.

Proper formation of the MHC peptide-loading complex is required for effective peptide loading in the ER and subsequent expression of MHC class I molecules at the cell surface [33]–[35]. Thus, regardless of the recovery of the peptide supply in the ER with coexpression of UL18 and US6, down-regulation of MHC class I at the cell surface might represent a defect in the formation of the MHC peptide-loading complex. We initially examined whether the expression of components of the peptide-loading complex is affected by UL18 or UL18/US6. Expression of UL18 or US6, individually or in combination, did not affect the expression level of MHC class I, TAP1, TAP2, or tapasin as evidenced by western blotting of whole cell lysates (Fig. 6*A*). Since assembly of the peptide-loading complex involves physical associations among components of the peptide-loading complex, we tested whether UL18 interfered with associations among components of the peptide-loading complex. HeLa cells infected with vvWT or vvUL18 were lysed in 1% digitonin. Cell lysates were analyzed by immunoprecipitation and western blot. Coimmunoprecipitation experiments revealed a reduced interaction between MHC class I and TAP1 or tapasin in the presence of UL18 alone (Fig. 6*B*). Interestingly, in the presence of US6, the ability of UL18 to perturb the association between MHC class I and TAP1 or tapasin was further enhanced (Fig. 6*C*). UL18 alone, or together with US6, did not affect the association of tapasin and TAP1 (Fig. 6*D*). These results indicate that UL18 disrupts an interaction specifically at the MHC-TAP1 and MHC-tapasin interfaces, but not at the tapasin-TAP1 interface. Given the importance of the MHC class I/tapasin/TAP interaction in optimal peptide loading of MHC class I molecules [36],[37], these results raise the possibility that UL18 inhibits optimal peptide loading of MHC class I molecules by competitively binding TAP.

## Discussion

HCMV encodes multiple proteins that down regulate the surface expression of MHC class I molecules [38]. This down-regulation allows infected cells to evade recognition by CTL but renders them susceptible to NK cells. HCMV encodes the UL18 protein, an MHC class I homolog that may protect infected cells from NK-mediated attack [19]. Despite the sequence and structural similarities between MHC class I molecules and UL18, the reason why UL18 molecules are not affected by US6 remains unclear [23]. In this study, UL18 induced dissociation of US6 from TAP1 to restore TAP-mediated peptide transport. UL18 also impaired optimal peptide loading onto MHC class I molecules by blocking the assembly of the MHC class I peptide-loading complex. Therefore, in the presence of UL18 and US6, surface levels of MHC class I molecules remained down regulated in spite of TAP function recovery.

The folding, assembly, and regulation of UL18 remain largely uncharacterized. UL18 loads peptides from endogenous protein precursors [22]. However, this precursor-peptide relationship does not distinguish between TAP-dependent or -independent peptide presentation. Our data demonstrate that normal surface expression of UL18 requires TAP function, as evidenced by the reduced surface expression of UL18 in TAP-deficient T2 cells (Fig. 1*C*). This TAP dependency was further confirmed by the down-regulation of UL18 surface expression by HSV-derived cytosolic TAP-inhibitor ICP47 [24],[25] (Fig. 1*B*).

**Figure 7:**
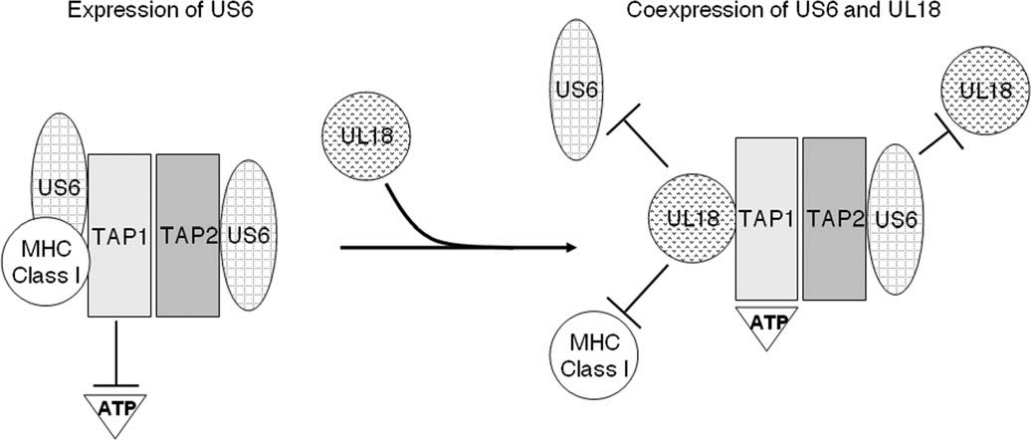
Hypothetical model for immune regulation of UL18 in the presence of US6. US6 binds to both TAP1 and TAP2, resulting in inhibition of ATP binding by TAP1 and, thereby, blocking TAP-mediated peptide translocation. Upon coexpression of UL18 and US6, TAP1 preferentially binds to UL18 relative to US6 and MHC class I, counteracting the US6-mediated inhibition of TAP. US6 preferentially binds to TAP2 over UL18, and thus, the US6-TAP2 interaction remains unaffected by UL18. As a result, even in the presence of ‘self-derived’ US6 TAP inhibitor, UL18 acquires peptides for assembly, whereas MHC class I molecules fail to load peptides due to incorrect assembly of the peptide-loading complex, despite the recovery of TAP function.

Our data provide an example of how the immune evasion gene products of HCMV have developed delicate mechanisms to avoid ‘self-attack’ and specifically target host proteins. Despite the TAP-dependency of UL18 for surface expression, surface expression of UL18 was insensitive to the self-derived TAP inhibitor US6 (Fig. 1). In contrast, the nonself-derived TAP inhibitor ICP47 down regulated surface expression of UL18. UL18 attenuated the inhibitory effect of US6 on TAP, resulting in the recovery of TAP function. UL18 then utilized the TAP-transported peptides for binding and subsequent surface expression. Given that UL18 inhibited the interaction between US6 and TAP1, but not between US6 and TAP2 (Fig. 3*B*), and that UL18 interacted with TAP1 (Fig. 3, *C* and *D*), an explanation for reversal of US6-mediated inhibition of TAP by UL18 might be that binding of UL18 to TAP1 causes conformational changes in TAP1 inducing dissociation from US6. The luminal domain of US6 associated with TAP [10]–[12], and under our experimental conditions, we did not detect the association of UL18 with US6. Thus, dissociation of US6 from TAP1 seems to occur indirectly as a consequence of UL18 binding to TAP1. Since US6 inhibits ATP binding to TAP1 but does not affect ATP binding to TAP2 [12], dissociation of US6 from TAP1 by UL18 would sufficiently restore TAP function. Our data show that TAP1 exhibits a preference for binding UL18 over US6 (Fig. 4, *C* and *D*). In contrast, TAP2 preferentially binds US6 over UL18 (Fig. 3, *B* and *D*). This differential affinity might be the molecular basis by which UL18 specifically modulates only the US6-TAP1 but not US6-TAP2 interaction (Fig. 7). The functional importance of physical association between TAP2 and US6 is not yet clear.

Interestingly, surface expression of MHC class I molecules was not recovered although the peptide supply was recovered upon coexpression of US6 and UL18 (Fig. 4*A*). Down-regulation of MHC class I in cells expressing UL18 alone was consistently observed in repeated independent experiments (Fig. 4*B* upper panel), although to a much lesser extent compared with the reduction levels induced by the US2, US3, US6, and US11 gene products [11],[23]. This finding is consistent with the observation that surface MHC class I expression is slightly increased in cells infected with UL18-deleted HCMV AD169. Depletion of the β_2_m pool by UL18 is not likely a mechanism for reducing the surface expression of MHC class I molecules, because high levels of free β_2_m are observed in HCMV-infected cells [39]. Instead, our findings suggest that UL18 and US6 interfere with the assembly of the MHC peptide-loading complex, despite the recovery of peptide supply upon coexpression of UL18 and US6. UL18 inhibited the physical interaction of MHC class I with both TAP1 and tapasin (Fig. 6, *B* and *C*) that is required for peptide loading and subsequent expression of MHC class I molecules on the cell surface, while the interaction between tapasin and TAP1 was not affected by UL18 (Fig. 6*D*). Given that tapasin is a known TAP stabilizer [40], preserving the tapasin-TAP interaction may allow UL18 to acquire peptide ligands. Due to the ability of UL18 to modulate US6, UL18 may still acquire peptides and serve as an inhibitory ligand for NK cells on the infected cell surface. In concert, UL18 might collaborate with US6 to disrupt assembly of the peptide-loading complex for CTL evasion.

The current findings may be of physiological relevance in the context of HCMV infection, if the temporal expressions of UL18 and US6 coincide during infection. US6 mRNA is expressed 8 to 120 h postinfection, and UL18 mRNA is detected later, from 54 to at least 120 h postinfection [23], indicating that indeed UL18 coexists with US6. Recently, surface-expressed UL18 on HCMV-infected fibroblasts was detected during this time frame [30]. As not all MHC class I alleles require the TAP-scaffolded peptide-loading complex for peptide loading [41], dissociation of MHC class I from TAP by UL18 might not be sufficient for evading CTL recognition. HCMV encodes a series of US gene products that function to down regulate the cell surface expression of MHC class I [42],[43]. Compared to the degree of MHC class I down-regulation by the US gene products, the effect of UL18 alone on MHC class I down-regulation was negligible (Fig. 4, *B* and *C*). Therefore, evading CTL immunity appears not to be the natural function of UL18. Instead, the physiological role of UL18 in modulating CTL recognition might be important, particularly in the context of US6 expression. Our study shows that UL18 utilizes US6 for its surface expression without compromising the ability of US6 to inhibit the cell surface expression of MHC class I. Thus, UL18 is a unique protein that has evolved to evade both the NK and the T cell immune response.

## Materials and Methods

### Cells, transfection, and viruses

HeLa cells were obtained from the American Type Culture Collection (ATCC, Manassas, VA) and cultured in DMEM (Life Technologies, Gaithersburg, MD) supplemented with 10% FBS (HyClone Laboratories, Logan, UT), 2 mM L-glutamine, 50 U/ml penicillin, and 50 µg/ml streptomycin. Human foreskin fibroblast (HFF) cells (passage 8–10) were grown in DMEM supplemented with 10% FBS under 5% CO_2_ at 37°C. Stable transfectants expressing tetracycline-inducible US6 (HeLa-US6) were described previously [11]. Transient transfection was performed using Lipofectamine (Invitrogen) according to the manufacturer's instructions. Recombinant vaccinia virus expressing UL18 was produced by cloning cDNA encoding the respective gene behind the early/late vaccinia virus p7.5 promoter into a modified pSC11 plasmid as previously described [44], and each plaque was purified three times in thymidine kinase-deficient 143B cells under bromodeoxyuridine selection (50 µg/ml). Cells were infected with recombinant vaccinia viruses at a multiplicity of infection of 10 for 1 h at 37°C in 500 µl of PBS supplemented with 10% BSA (Sigma-Aldrich, St. Louis, MO).

### Antibodies

mAb 10C7 recognizing UL18 was purchased from the ATTC. K455 recognizes MHC class I heavy chain and β_2_m in both assembled and nonassembled forms [45]. Anti-MHC class I polyclonal Ab H-300 (sc-25619) was purchased from Santa Cruz Biotechnology (Santa Cruz, CA). Normal mouse IgG was purchased from Sigma-Aldrich. Polyclonal antisera specific for US6, human TAP1, and TAP2 raised against synthetic peptides have been described [11],[46]. Anti-CD59 antibodies were obtained from BD Biosciences (Mountain View, CA).

### Flow cytometry

The surface expression of UL18 and MHC class I molecules was determined by flow cytometry (FACSCalibur; BD Biosciences, Mountain View, CA). Cells were washed twice with cold PBS containing 1% BSA and then incubated for 1 h at 4°C with either 10C7 for UL18 or W6/32 for MHC class I molecules. Normal mouse IgG was used as a negative control. The cells were washed twice with cold PBS containing 1% BSA and then stained with FITC-conjugated goat anti-mouse IgG for 40 min. A total of 10,000 gated events were collected by the FACSCalibur cytometer and analyzed with CellQuest software (BD Biosciences).

### Coimmunoprecipitation and immunoblotting

For coimmunoprecipitation, cells were lysed in 1% digitonin in PBS supplemented with protease inhibitors. After preclearing, samples were incubated with the appropriate antibodies for 2 h at 4°C, before Protein G-Sepharose beads were added. Beads were washed four times with 0.1% digitonin, and bound proteins were eluted by boiling in SDS sample buffer. Proteins were separated by SDS-PAGE, transferred onto a nitrocellulose membrane, blocked with 5% skim milk in PBS with 0.1% Tween 20 for 2 h, and probed with the appropriate antibodies for 4 h. Membranes were washed three times in PBS with 0.1% Tween 20 and incubated with horseradish peroxidase-conjugated streptavidin (Pierce) for 1 h. The immunoblots were visualized with ECL detection reagent (Pierce).

### Peptide transport, ATP-agarose binding, and thermostability assays

The peptide transport assay was performed as described [42]. HeLa and HeLa-US6 cells infected with vvWT or vvUL18 were permeabilized with Streptolysin-O for 20 min at 37°C and incubated for 10 min at 37°C in 5 µM of an FITC-conjugated peptide (RYNATGRL) in the presence of 1 mM DTT and 10 mM ATP. Following lysis in 1% NP-40, the glycosylated peptide fraction was isolated using ConA-Sepharose beads, and fluorescence of the peptides eluted from ConA was measured using a fluorescence reader (HTS 7000 Bio Assay Reader; Perkin-Elmer, Norwalk, CT). The ATP-agarose binding assay was performed as described [12]. Cells infected with either vvWT or vvUL18 were used for analysis. Thermostability assays were performed essentially as described [32].
